# Endoscopic stenting for hilar cholangiocarcinoma: efficacy of unilateral and bilateral placement of plastic and metal stents in a retrospective review of 480 patients

**DOI:** 10.1186/1471-230X-12-103

**Published:** 2012-08-09

**Authors:** Manuel José Antunes Liberato, Jorge Manuel Tavares Canena

**Affiliations:** 1 Center of Gastroenterology, Hospital Cuf Infante Santo, Travessa do Castro n º 3, 1350-070, Lisbon, Portugal

**Keywords:** Hilar cholangiocarcinoma, Endoscopic palliation, Plastic stents, Self-expandable metal stents, Bilateral stenting

## Abstract

**Background:**

Endoscopic biliary drainage of hilar cholangiocarcinoma is controversial with respect to the optimal types of stents and the extent of drainage. This study evaluated endoscopic palliation in patients with hilar cholangiocarcinoma using self-expandable metallic stents (SEMS) and plastic stents (PS).We also compared unilateral and bilateral stent placement according to the Bismuth classification.

**Methods:**

Data on 480 patients receiving endoscopic biliary drainage for hilar cholangiocarcinoma between September 1995 and December 2010 were retrospectively reviewed to evaluate the following outcome parameters: technical success (TS), functional success (FS), early and late complications, stent patency and survival. Patients were followed from stent insertion until death or stent occlusion. Patients were divided into 3 groups according to the Bismuth classification (Group 1, type I; Group 2, type II; Group 3, type > III).

**Results:**

The initial stent insertion was successful in 450 (93.8%) patients. TS was achieved in 204 (88.3%) patients treated with PS and in 246 (98.8%) patients palliated with SEMS (p < 0.001). In the intention-to-treat (ITT) analysis, the FS in patients treated with SEMS (97.9%) was significantly higher than in patients treated with PS (84.8%) (p < 0.001). Late complications occurred in 115 (56.4%) patients treated with PS and 60 (24.4%) patients treated with SEMS (p < 0.001). The median duration of stent patency in weeks (w) were as follows: 20 w in patients palliated with PS and 27 w in patients treated with SEMS (p < 0.0001). In Group 2, the median duration of PS patency was 17 w and 18 w for unilateral and bilateral placement, respectively (p = 0.0004); the median duration of SEMS patency was 24 w and 29 w for unilateral and bilateral placement, respectively (p < 0.0001). Multivariate analysis using the Poisson regression showed that SEMS placement (B = 0.48; *P* < 0.01) and bilateral deployment (B = 0.24; *P* < 0.01) were the only independent prognostic factors associated with stent patency.

**Conclusions:**

SEMS insertion for the palliation of hilar cholangiocarcinoma offers higher technical and clinical success rates in the ITT analysis as well as lower complication rates and a superior cumulative stent patency when compared with PS placement in all Bismuth classifications. The cumulative patency of bilateral SEMS or PS stents was significantly higher than that of unilateral SEMS or PS stents, with lower occlusion rates in Bismuth II patients.

## Background

Cholangiocarcinoma (CCA) is the primary cancer of the bile ducts and it arises from the malignant transformation of cholangiocytes, which are the epithelial cells that line the biliary apparatus. Although it comprises only 10-15% of hepatobiliary neoplasms, the incidence of CCA has increased during the past 3 decades [1]. Hilar CCA was first recognized as a distinct clinical entity in 1965 when Klatskin [2] reported on a series of 13 patients. Malignancies of the biliary hilum have an extremely poor prognosis, with a 5-year survival rate of less than 10% [3]. In the majority of cases, curative resection is not a therapeutic option, and palliation is the main goal of therapy [3,4]. The relief of biliary obstruction not only reduces jaundice and associated pruritus but also improves related symptoms such as anorexia and disturbed sleep patterns and leads to an improved quality of life [5]. Endoscopic biliary drainage is preferable to percutaneous or surgical procedures and has become the standard of care because of its low invasiveness [4-7].

Endoscopic palliative bile duct drainage was first reported by [Soehendra et al. 8]. Currently, two types of endoscopic stents are available. With the introduction of duodenoscopes with 4.2-mm working channels in 1982, the endoscopic insertion of large-bore plastic biliary stents (PS) became possible [9]. The main disadvantage of plastic endoprostheses is a relatively high occlusion rate caused by biliary sludge, which occurs at a median interval of 3 to 4 months after placement [3]. First described in 1989 [10,11], self-expandable metal stents (SEMS) are available with different lengths, diameters and delivery devices. SEMS with a maximum diameter of 10 mm theoretically offer the optimal conditions for long-term drainage; however, only two prospective randomized trials have compared PS with SEMS in the management of patients with hilar biliary obstruction [12,13]. In an older study, Wagner et al. reported that SEMS placement resulted in higher long-term patency rates and higher technical success rates with diminished costs [12]. A recent randomized controlled trial has just been published, which demonstrated superior outcomes in patients treated with metal stents both in terms of success of drainage and survival [13]. One study [14] reviewed a small number of consecutive patients who underwent SEMS or PS placement for hilar obstruction and also reported that SEMS yielded better outcomes. A recent paper [15] comparing the clinical effectiveness of SEMS and plastic stents in patients with inoperable hilar CCA showed a significantly better stent patency for all Bismuth classifications [16] when metal stents were used. However, few comparative data are available from a large number of patients to address whether PS or SEMS is preferable for palliating malignant hilar obstruction, and it remains unclear whether or not SEMS placement is superior to plastic stent drainage.

The benefit of bilateral versus unilateral stenting continues to be debated [17-22]. Only one randomized controlled study using PS has compared unilateral with bilateral drainage [20], and the authors concluded that the insertion of more than one endoprostheses did not appear to be justified as a routine procedure and that single-stent insertion was effective. However [Deviére et al. 21] using two or more PS to achieve complete drainage of the biliary system showed that this approach improves survival and reduces procedure-related mortality and the incidence of early and late cholangitis when compared to incomplete drainage. Thus far, no prospective randomized study has compared the unilateral and bilateral placement of SEMS. A recent study suggested more favorable cumulative stent patency with bilateral over unilateral SEMS placement in cases of hilar obstruction, especially in cases of CCA [21]. The endoscopic deployment of bilateral SEMS is technically challenging, even for experienced endoscopists. Various techniques have been described for bilateral SEMS placement [5,17,22-28]. The most commonly used technique is the stent-within-stent method, in which a wide mesh SEMS (although a stent with a closed-cell configuration can also be used) is inserted into one side of the hepatic duct, and a second SEMS is positioned on the contralateral side across the mesh [23,24]. Recently, SEMS with extra-wide open mesh designs in the central portion to facilitate bilateral placement have been described, and the initial trials have shown encouraging results [25,26]. Other studies have described techniques to place the SEMS in a side-by-side configuration, with good results [5,17,22]. Recently, a novel SEMS was developed with a 6 French delivery system to allow a side-by-side pre-deployment insertion of bilateral SEMS [27].

The aim of this article is to report on the experience of our single tertiary-level center in the endoscopic palliation of hilar CCA during the last 15 years. In this study, we compared the technical success, functional success, early complications, late complications, stent patency and survival for patients treated with PS and SEMS. We also compared unilateral and bilateral stent placement according to the Bismuth classification.

## Methods

### Patients and study design

This was a retrospective review of cases of endoscopically inserted plastic and metal stents in patients with unresectable hilar CCA over a period of 15 years (September 1995 to December 2010). Data were retrospectively reviewed from a dedicated prospective computerized endoscopy database at a tertiary referral academic center (Center of Gastroenterology, Cuf Infanto Santo Hospital, Lisbon, Portugal). The diagnosis was based on ultrasound, computed tomography, magnetic resonance cholangiography, endoscopic retrograde cholangiopancreatography (ERCP), clinical history and histologic or cytologic confirmation by pathologists. Histologic confirmation of cholangiocarcinoma was obtained in 82 patients (17%) by histopathologicevaluation of cytology (65 patients) or a biopsy of the hilar lesion (17 patients). In patients without histological confirmation the diagnosis was based on imaging and clinical outcome during follow-up. Patients were considered to have an unresectable malignancy based on their medical fitness (57/480 patients - 11.9%) or disease extent (423/480 patients - 88.1%). The follow-up continued from stent insertion until patient death or stent occlusion. The endoscopic and medical reports of these patients were reviewed together with the radiological findings to determine the outcome in terms of the following parameters: technical success, functional success, early and late complications, stent patency and survival. Hilar obstructions were characterized by the Bismuth classification [16].

Patients were divided into 3 groups: Group 1 included patients with a type I malignant stricture, Group 2 included patients with a Bismuth type II cancer and Group 3 included patients with Bismuth type III or IV strictures. Patients in Group 1 were further divided into Group 1A if they were palliated with PS or Group 1B if they underwent SEMS placement. Patients in Group 2 had unilateral or bilateral placement of PS or SEMS and were divided in 4 subgroups: Group 2A was palliated with 1 PS, Group 2B was palliated with bilateral PS, Group 2C underwent 1 SEMS placement and Group 2D underwent bilateral SEMS placement. Patients in Group 3 were divided into Group 3A if they underwent PS placement or Group 3B if they received an SEMS. The intention-to-treat (ITT) and per protocol (PP) methods were used in this analysis. The ITT analysis was based on the original total cohort of patients enrolled. The PP analysis was based on the subset of patients with successful unilateral or bilateral stent placement. Patient characteristics and stent insertions were evaluated by the ITT analysis. Stent patency, complications and survival were evaluated by the PP analysis. All patients provided full, informed written consent prior to their procedures. The study was approved by our institutional review board.

### Definition of events

Technical success (TS) or successful stent insertion was defined as the passage of the stent across the stricture along with the flow of contrast medium and/or bile through the stent. Functional success (FS) or successful drainage was defined as a decrease in bilirubin to less than 75% of the pre-treatment level within the first month. Early and late complications were defined as those occurring within 30 days and after 30 days of stent placement, respectively, according to the criteria of [Cotton et al. 29]. Occlusion of the stent was considered if patients had recurrent jaundice with cholestasis or cholangitis (fever, increase in serum bilirubin, leucocytosis) and dilatation of intrahepatic bile duct, revealed by imaging. Stent patency was defined as the period between stent insertion and stent occlusion or patient death.

### Technique

A prophylactic treatment with broad-spectrum antibiotics was initiated before the procedure and continued for 10 days after. All procedures were performed by 2 experienced pancreatobiliary endoscopists (M.L. and J.C.). ERCP procedures were performed with the patient in the prone position under sedation, with propofol administered by an anaesthesiologist. All endoscopic examinations were performed using a therapeutic duodenoscope (TJF-145, TJF-160R, TJF-160VR, Olympus, Tokyo, Japan). The strategy used for biliary decompression was selected at the discretion of the endoscopist, based on the location and complexity of the strictures as well as the expected time of survival of the patient. Plastic stents were not changed routinely, and patients were treated if occlusion of the stent or cholangitis developed. Unilateral PS stenting was done with 10 French stents. If the patient had bilateral PS placed, at least one of the stents had a diameter of 10 French. Decision on unilateral versus bilateral endoscopic drainage was left to the discretion of the endoscopist based on location and complexity of the strictures. Biliary sphincterotomy was performed in all cases to facilitate single or bilateral stent placement.

**Figure 1:**
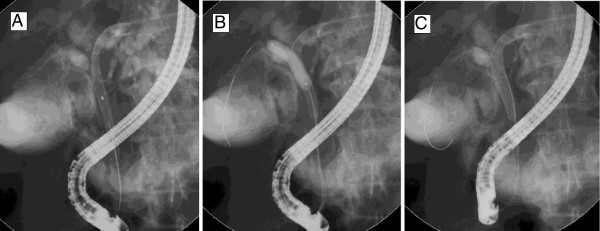
**Bilateral self-expandable metal stents (SEMS) placement using a stent-within-stent technique.** (**A**) After deployment of the first stent across the hilar stricture, the guidewire was inserted, under fluoroscopic guidance, into the contralateral hepatic duct trough the interstices of the initial SEMS. (**B**) Dilatation of the interstices of the first SEMS with a hydrostatic balloon to facilitate the passage of a second SEMS into the contralateral hepatic duct. (**C**) Bilateral SEMS placement obtaining a Y-shaped configuration.

**Figure 2:**
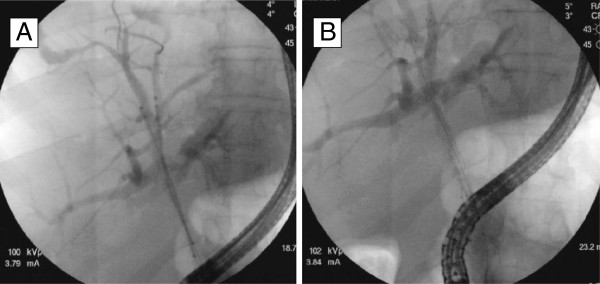
**Bilateral metal stenting using the novel 6 French delivery system.** (**A**) Side-by-side bilateral hilar predeployment. (**B**) Bilateral hilar postdeployment to form a Y-shaped configuration.

**Figure 3:**
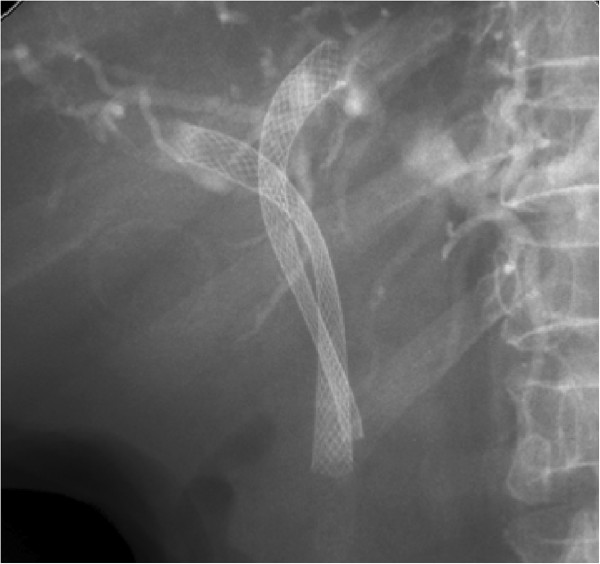
Bilateral metal stenting using side-by-side deployment.

For bilateral SEMS placement, we routinely use a previously described stent-within-stent technique (Figure 1) [23,24]. In a small subset of patients, bilateral drainage with SEMS was achieved using 2 different side-by-side techniques. In the first technique, as described in detail elsewhere [27], we used a novel non-foreshortening metal stent with a 6 French delivery system (Zilver 635; Cook Medical, Winston-Salem, NC) to enable side-by-side bilateral hilar deployment because duodenoscopes with 4.2-mm working channels allow the simultaneous delivery of both stents. After bilateral guidewire placement, the Zilver 635 biliary uncovered metal stent deployment system was sequentially introduced over the guidewires into the left and right hepatic ducts in a side-by-side fashion. Biliary dilatation was performed as needed to facilitate stent placement. The stents were then carefully deployed across the strictures in alternating/sequential fashion between the left and right sides until full deployment was achieved (Figure 2). All SEMS used had a 10-mm luminal diameter and an 80-mm length. In the second technique, we also performed placement of bilateral stents in a sequential fashion [5,17,22]. After the insertion of bilateral guidewires, we performed bilateral balloon dilatation of the stenosis. The first SEMS was usually placed in the left hepatic duct. The second SEMS was advanced to the right hepatic duct after the expansion of the initial stent. Stents are usually placed with their distal ends at the same level intraductally, i.e., not in a transpapillary position (Figure 3). Special care was taken to place both SEMS at the same level to facilitate re-entry in future if needed. 

### Follow-up

Follow-up continued from stent insertion to the death of the patient or to the stent occlusion for all patients. Only patients in whom the stent occlusion was reviewed in our center were included in the study. Follow-up was obtained by reviewing clinical notes provided by regular clinic visits or by medical assistant reports, lab results, imaging, data on our prospective database and structured telephone interviews with medical assistants or family during follow-up or at the time of manuscript preparation. Patients with incomplete follow-up were excluded from the study. All of the patients were dead at the time of manuscript preparation.

### Statistical analysis

Student’s *t* test and the *χ*^2^ test were used to calculate the statistical significance of different demographic and clinical variables when appropriate. Cumulative stent patency and cumulative patient survival were calculated by the Kaplan-Meier method, and groups were compared by the log-rank test. Multivariate analysis was conducted using a Poisson regression to determine possible factors affecting stent patency. Age, sex, type of stent and type of deployment (unilateral vs. bilateral stent placement) were the variables included in the analysis. All reported *P*-values were for two-sided test and a *P-*value less than 0.05 was considered to be statistically significant. Statistical analysis was performed by using SPSS (Statistical Package for the Social Sciences) 18 (IBM Corporation, New York, USA) and STATA v11.0 (StataCorp Lp, College Station, Texas, USA).

## Results

### Patients

A total of 12850 records for ERCP procedures that occurred between September 1995 and December 2010 were identified in the endoscopy database. In total, 528 records for patients with hilar CCA who underwent endoscopic insertion of plastic and metal stents were selected in the endoscopy database. Forty-eight of these patients were excluded for the following reasons: incomplete data concerning endoscopic procedures and follow-up (37 patients) and further surgery after the initial stenting (11 patients).

In total, 480 patients (249 male and 231 female) with a mean age of 74.6 years (range: 45–96) were enrolled in the study. There were 231 and 249 patients palliated with PS and SEMS, respectively. The characteristics of the patients are shown in Table 1. There were no significant differences in the demographics between patients treated with PS and those treated with SEMS or for unilateral and bilateral stenting, overall and among the groups defined earlier in the Methods section.

**Table 1:** Patient characteristics

	**Plastic stent**	**Metal stent**	***P*** **value**
Group 1	N = 115	N = 99	
Sex (%)	60 M (52.2), 55 F (47.8)	50 M (50.5), 49 F (49.5)	0.8081
Age (years)	73.9 (45–88)	74.3 (50–91)	0.7462
Group 2	N = 67	N = 78	
Sex (%)	30 M (44.8), 37 F (55.2)	39 M (50), 39 F (50)	0.5301
Age (years)	74.9 (49–93)	74.7 (51–95)	0.8912
Group 3	N = 49	N = 72	
Sex (%)	30 M (61.2), 19 F (38.8)	40 M (55.6), 32 F (44.4)	0.5351
Age (years)	75.4 (58–94)	75.1 (56–90)	0.8402
Total	N = 231	N = 249	
Sex (%)	120 M (51.9), 111 F (48.1)	129 H (51.8), 120 M (48.2)	0.9751
Age (years)	74.5 (45–94)	74.7 (50–95)	0.8262

M, male; F, female; Group 1, patients with Bismuth type I malignant strictures; Group 2, patients with Bismuth type II malignant strictures, Group 3, patients with Bismuth type III and IV malignant strictures; Age in mean (range).

### Stent insertion

The outcomes of the stent insertion are shown in Tables 2 and 3. The initial stent insertion (Table 2) was successful in 450 (93.8%) patients. TS was achieved in 204 (88.3%) and 246 (98.8%) patients palliated with PS and SEMS, respectively. There were 30 patients with failed endoscopic endoprostheses placement. Among these patients, 10 received percutaneous drainage, 4 underwent surgery and the remaining 16 were considered unfit for further interventions and transferred to palliative care. FS was achieved in 440 patients (ITT = 91.6%; PP = 97.8%). All patients with failed successful drainage were in Group 3 (8 in Group 3A and 2 in Group 3B). These patients showed no improvement of bilirubin after the stent placement because of extensive hepatic involvement. Five of these patients underwent bilateral percutaneous biliary drainage without significant reductions in the bilirubin level. The remaining 5 patients had significant comorbidities and very advanced disease, and they were not candidates for further intervention. SEMS comparison with PS provided significantly different degrees of technical success, overall (*P* < 0.001) and in Group 1 (*P* = 0.036), Group 2 (*P* = 0.008) and Group 3 (*P* < 0.001). In the ITT analysis, the FS in patients treated with SEMS was significantly higher than that in patients treated with PS overall (*P* < 0.001) and in Group 1 (*P* = 0.036), Group 2 (*P* = 0.008) and Group 3 (*P* < 0.001). In the PP analysis, the functional success in patients treated with SEMS was significantly higher than that in patients treated with PS both overall (*P* = 0.026) and in Group 3 (*P* < 0.001).

**Table 2:** Stent insertion outcome

			**Plastic**			**Metal**			***P***
		**n**	**TS**	**FS**	**n**	**TS**	**FS**	**TS**	**FS**
	n	115	110	110	99	99	99		
Group 1	ITT (%)		(95.7)	(95.7)		(100)	(100)	0.036	0.036
	PP (%)			(100)			(100)		-
	n	67	59	59	78	77	77		
Group 2	ITT (%)		(88.1)	(88.1)		(98.7)	(98.7)	0.008	0.008
	PP (%)			(100)			(100)		-
	n	49	35	27	72	70	68		
Group 3	ITT (%)		(71.4)	(55.1)		(97.2)	(94.4)	<0.001	<0.001
	PP (%)			(77.1)			(97.1)		0.001
	n	231	204	196	249	246	244		
Total	ITT (%)		(88.3)	(84.8)		(98.8)	(97.9)	<0.001	<0.001
	PP (%)			(96.1)			(99.2)		0.026

ITT, intention-to-treat; PP, per protocol; TS, technical success; FS, functional success; Group 1, patients with Bismuth type I malignant strictures; Group 2, patients with Bismuth type II malignant strictures; Group 3, patients with Bismuth type III and IV malignant strictures.

**Table 3:** Stent insertion outcome in Group 2

		**Plastic stent**		**Metal stent**	
	Unilateral (n)		21		35
Group 2 (n)		59		77	
	Bilateral (n)		38		42

^a^Group 2, patients with Bismuth type II malignant strictures. Unilateral and bilateral stenting with SEMS and PS.

Stent insertion outcomes for Group 2 are shown in Table 3. Bilateral stenting was performed in 38 patients with PS and in 42 patients with SEMS. Bilateral PS was attempted in 40 patients, and stents were successfully placed in 38 patients (95%). The remaining 2 patients had unilateral PS placement. Failure to place the guidewire in the contralateral duct was the reason for failure in these 2 patients. Bilateral SEMS placement was attempted in 45 patients, and successful bilateral drainage was achieved in 42 (93.3%) patients. The remaining 3 patients were treated with unilateral SEMS. The main reason for unsuccessful bilateral SEMS insertion was a failure to place the guidewire into the contralateral hepatic duct through the interstices of the initial SEMS. The stent-within-stent technique was used in 36/42 patients (85.7%), whereas the side-by-side stent placement was used in 6/42 patients (14.3%). There were no significant differences in the ITT and PP analyses of TS and FS in patients with unilateral vs. bilateral PS and SEMS placement.

### Complications and reinterventions

#### Early complications

Early complications (Table 4) occurred in 17 (8.3%) patients treated with PS and in 5 (2.0%) patients who underwent SEMS placement (*P* = 0.002). Early complications included pancreatitis, bleeding and stent failure (occlusion and migration). There was no procedure-related mortality.

**Table 4:** Complications

	**Plastic (n=204)**		**Metal (n=246)**		***P*** **value**	**Total**	
	**n**	**%**	**n**	**%**		**n**	**%**
*Early Complications*	17	8.3	5	2	0.002^a^	22	4.9
Stent failure	12	5.9	0	0	<0.001^a^	12	2.7
- occlusion	8	3.9	0	0	0.017^a^	8	1.8
- migration	4		0	0	0.027^a^	4	0.9
Pancreatitis	4	2.0	3	1.2	0.534	7	1.6
Bleeding	1	0.5	2	0.8	0.670	3	0.7
*Late Complications*	115	56.4	60	24.4	<0,001^a^	175	38.9
Stent failure	115	56.4	60	24.4	<0,001^a^	175	38.9
- occlusion	106	52.0	60	24.4	<0,001^a^	166	36.9
with cholangitis	68/106	33.3	14/60	5.7	<0,001^a^	83/166	18.2
- migration	9	4.4	0	0	0,001^a^	9	2.0

^a^Statistically significant.

#### Late complications

Late complications (Table 4) occurred in 115 (56.4%) patients treated with PS and in 60 (24.4%) patients treated with SEMS, and these differences were significant (*P* < 0.001). All late complications were stent-related and included migration (only in PS patients), occlusion and cholangitis. There were significant differences in occlusion and cholangitis when comparing patients treated with PS and those treated with SEMS. Repeat endoscopic biliary drainage because of stent failure was required in 56.4% of patients with PS. In the group of patients with SEMS, 75.6% did not require any further intervention. In Group 2, 17/21 (80.9%) patients with unilateral PS placement and 13/38 (34.2%) patients treated with bilateral PS stenting (*P* < 0.001) had stent occlusion, respectively. Repeat endoscopic biliary drainage because of stent occlusion was required for 11/35 (31.4%) patients treated with one SEMS versus 5/42 (11.9%) patients with bilateral SEMS placement (*P* = 0.036).

More reinterventions were required in the PS group (n = 127) than in the SEMS group (n = 60) for stent failure and these differences were statistically significant (*P* < 0.001).

### Stent patency

**Figure 4:**
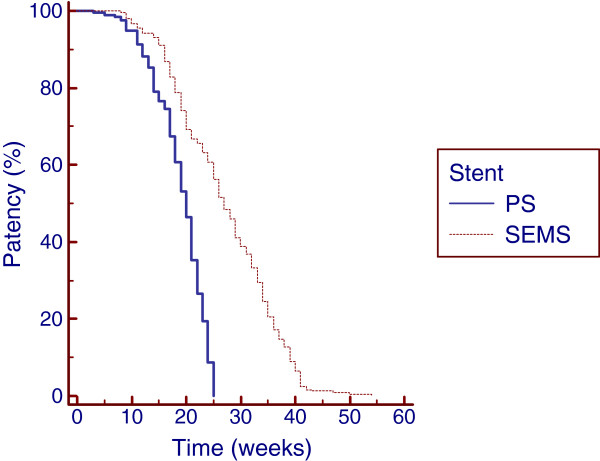
Kaplan-Meier analysis showing that cumulative stent patency was significantly longer in patients treated with self-expandable metal stents (SEMS) than in patients palliated with plastic stents (PS) (p < 0.0001).

**Figure 5:**
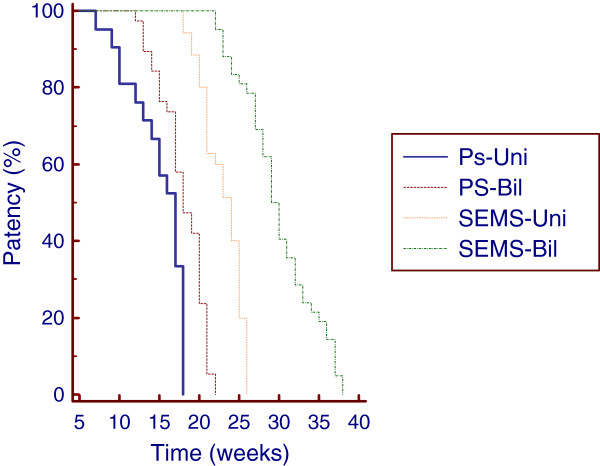
**Cumulative Stent Patency curves by Kaplan-Meier analysis in patients treated using unilateral (Uni) and bilateral (Bil) plastic stents (PS) and self-expandable metal stents (SEMS).** Uni PS versus Bil PS (p = 0.0004). Uni versus Bil SEMS (p < 0.0001).

Patients treated with PS and SEMS had a median time to occlusion of 20 weeks (range: 3–25; mean: 18.9) and 27 weeks (range: 8–54; mean: 27.1), respectively. Kaplan-Meier analysis showed that the cumulative stent patency times were significantly longer in patients treated with SEMS than in those treated with PS (*P* < 0.0001); the estimated relative risk of occlusion (hazard ratio-HR) was 2.6731-fold higher in the PS group than in the SEMS group, (95% CI 2.1416-3.3365) (Figure 4). The median stent patency time was 22 weeks (range: 14–25; mean: 22) in Group 1A and 35 weeks (range: 25–54; mean: 35) in Group 1B. These differences were significant (*P* < 0.0001); the HR was 4.6219 higher in Group 1A than in Group 1B, (95% CI 3.2799-6.5129). In Group 3A, stent occlusion occurred after a median time of 12 weeks (range: 3–14; mean: 11), whereas in Group 3B, the median time to occlusion was 17 weeks (range: 8–20; mean: 16) (*P* < 0.0001). The HR was 3.8195-fold higher in Group 3A than in Group 3B, (95% CI 1.9186-7.6037). The median stent patency time was 17 weeks (range: 7–18; mean: 15) in Group 2A (PS, unilateral) and 18 weeks (range: 12–22; mean: 18) in Group 2B (PS, bilateral). The median stent patency time was 24 weeks (range: 18–26; mean: 23) in Group 2C (SEMS, unilateral) and 29 weeks (range: 22–28; mean: 30) in Group 2D (SEMS, bilateral). Kaplan-Meier analysis showed a significant difference in the cumulative stent patency time for bilateral and unilateral plastic stenting (*P* = 0.0004), and the HR was 2.2355-fold higher in Group 2A than in Group 2B (95% CI 1.1787-4.2399) (Figure 5). The cumulative stent patency time for bilateral SEMS stenting was significantly longer than that for unilateral SEMS stenting (p < 0.0001), with an HR that was 3.6934 higher in Group 2C than in Group 2D (95% CI 2.0755-6.5724) (Figure 5). Multivariate analysis using the Poisson regression showed that SEMS placement (B = 0.48; *P* < 0.01) and bilateral deployment (B = 0.24; *P* < 0.01) were the only independent prognostic factors associated with stent patency.

### Survival

The median survival time was 46 weeks (range: 9–61; mean: 46.1) and 45 weeks (range: 7–59; mean: 44.9) in patients treated with PS and SEMS, respectively. Kaplan-Meier analysis showed no differences in the cumulative survival between the 2 groups (*P* = 0.18), with an HR 1.1777-fold higher in the SEMS group than in the PS group (95 % CI 0.9104-1.5236).

## Discussion

According to our findings, in a retrospective review of 480 patients, the palliation of hilar CCA with SEMS was associated with successful stent insertion and successful drainage in the ITT analysis as well as a lower complication rate and increased cumulative stent patency compared with PS placement for all Bismuth classifications. The outcome in terms of FS after PS insertion in patients with advanced disease (Group 3) was very poor. In a subset of patients with Bismuth type II hilar strictures, the cumulative stent patency of bilateral SEMS or PS placement was significantly higher than that of unilateral SEMS or PS stenting, with lower occlusion rates. In this group of patients, bilateral SEMS placement offered the best results in terms of cumulative stent patency and occlusion rates.

Our data results from a single academic tertiary center which is a referral center for ERCPs especially difficult cases and tumors. It is the largest center in the country receiving patients from 20–30 hospitals all over the southern part of the country. We perform an average of 850 ERCPs per year, thus having a huge inclusion potential as far as hilar CCAs are concerned. Furthermore our center is the main referral center for endoscopic palliation of malignancies, coming from oncology, gastroenterology and surgical centers.

There have been few comparative studies regarding PS and SEMS placement in hilar CCA, and because such data are scarce, no clear consensus has been reached regarding the optimal approach in these patients. A recent randomized trial randomly allocated 108 patients to SEMS or PS placement [13]. The authors reported that endoscopic biliary drainage with SEMS was associated with significantly successful drainage rate and longer survival compared with PS placement. [Raju et al. 15] recently presented a retrospective review of 100 patients who underwent PS and SEMS placement for inoperable hilar CCA. Patients were divided into 3 groups according to the Bismuth classification, similarly to our study. The SEMS group demonstrated a significantly higher patency (5.56 vs. 1.86 months) and required fewer re-interventions for stent obstruction. The authors concluded that the patency of metallic stents was superior to that of PS in all Bismuth classifications, although a clear comparison between the groups was not presented in the paper.

Our study suggests that patency of SEMS is significantly longer with lower occlusion rates when compared with PS. In our paper, the duration of patency in the plastic stent group was longer than previously reported [15,30]. This finding may be due to the result of the high number of patients with Bismuth type I strictures enrolled in our study, as PS placed in patients with more advanced disease tend to have shorter patency times [15,30]. The use of SEMS in hilar CCA has multiple theoretical advantages, including a small and flexible delivery system that allows for better technical success and flexibility and a mesh network that enables not only a more stable conformation with the tortuous hilar anatomy but also drainage of subsegmental ducts. In our study, we found better TS when using SEMS, which could be related to the inherent characteristics of the stents, as long and inflexible large-bore plastic stents are difficult to place in complex strictures. Overall, the TS was high in both groups (88.3% in the PS group and 98.8 % in the SEMS group), and these differences were significant. More complex strictures showed greater differences in TS when comparing PS with SEMS. Even in Group 1, there were significant differences between the two stent types (95.7% versus 100%), and this finding reflects our large sample size. As expected, the FS of PS placement in patients with more advanced disease (Group 3) was lowest, not only in the ITT analysis but also in the PP analysis. This finding could be the result of the lack of side holes in this type of stent, which can result in reduced drainage from secondary branch ducts. In our study, PS were not routinely changed, which was the main cause of the significantly higher rate of late complications associated with stent failure in patients treated with PS. Overall, our study suggests that PS stenting is associated with worse outcomes than SEMS placement; therefore, endoscopic palliation with PS should be reserved for pre-surgical biliary drainage when needed and for patients with a short expected survival time.

The optimal technique for endoscopic palliative metal placement and the benefits of bilateral versus unilateral stenting are still controversial and highly debated. [De Palma et al. 20] reported the only prospective randomized controlled study comparing unilateral and bilateral drainage using plastic stents in 157 patients. In the ITT analysis, unilateral placement had a significantly higher rate of stent insertion (88.6% vs. 76.9%) and a lower rate of complications and early cholangitis than bilateral placement. The authors concluded that the routine insertion of more than one stent would not be justified and that single stent insertion avoids the risk of further procedure-related complications and mortality. However these results need to be interpreted with caution because of some study biases. Information about stent patency and occlusion rates in both groups was not available. Furthermore subgroup analysis of patients was not done and there was a high number of patients with Bismuth type I stricture included for which placement of one stent is sufficient; thus it is impossible to find out how results might have been affected by their inclusion. A recent retrospective review of 46 patients with hilar malignant obstruction compared unilateral with bilateral SEMS stenting [22]. Cumulative stent patency was significantly increased with bilateral stenting (median patency of 488 days vs. 210 days, *P* = 0.009), especially in cases of CCA.

Endoscopic bilateral metal drainage poses particular challenges for endoscopists and has been considered to be more technically challenging than unilateral stenting [5,17,19,22-28,31]. Various techniques have been described for bilateral SEMS placement, and in the absence of a truly Y-shaped SEMS, the creation of a Y-shaped stent configuration across the hilar bifurcation requires the placement of straight SEMS in either a nested or parallel configuration. Most endoscopists use the stent-within-stent technique previously described [23,24] in this paper. Theoretically, stent-within-stent deployment may prevent bile influx into the area of stent overlap, leading to sludge formation. Furthermore, tumor ingrowth can occur more easily through an expanded stent mesh in the area of overlap. In addition, a nested SEMS configuration can be difficult to revise when cancer ingrowth obstructs the stents. To facilitate nested Y-shaped SEMS placement across the biliary confluence, SEMS placement with an extra-large open mesh in the central portion of the stent has been described. [Kim et al. 25] reported their experience in 34 patients, in which TS was achieved in 85.3% patients, and the FS in the PP analysis was noted to be 100%. Stent obstruction occurred in 31% of the patients, and the median duration of stent patency was 239 days.

Endoscopic bilateral metal stenting can also be accomplished using a parallel arrangement known as side-by-side deployment [5,17,22]. It has been reported that this technique occasionally causes portal vein occlusion and increases the rate of cholangitis because of the excessive expansion of the bile duct by parallel stents [22]. In some cases, this technique is impossible once one SEMS has been deployed, even if a second guidewire is already in the contralateral duct, because the first stent may press into the bile-duct wall and prevent passage of the delivery system for the second SEMS. One solution for this problem [5] is to place a temporary plastic stent in a sub-hilar position, with 2 guidewires placed in the right and left hepatic ducts, before deployment of the first SEMS. After the placement of the first SEMS beside the plastic stent, the delivery catheter for the second SEMS is advanced into the contralateral duct, with the plastic stent maintaining the passage. Following the deployment of the second SEMS, the plastic stent is removed. Another solution to side-by-side SEMS placement is to use the novel SEMS with a 6 F delivery system. Using this technique, [Chennat et al. 27] reported a TS of 100% (10/10 patients) and a median stent patency of 130 days. The side-by-side approach may facilitate subsequent endoscopic access to both drained ductal segments. A recent study [32] compared side-by-side versus stent-within-stent deployment in 52 consecutive patients with malignant hilar obstruction. The authors found no differences in TS and FS between groups. Side-by-side deployment was associated with a higher rate of complications and a significantly better stent patency in Kaplan-Meyer analysis but not on multivariate analysis. Overall is not clear that a technique is better than the other and further studies on this issue are needed.

In our study, bilateral stenting was only reported in patients with Bismuth type II strictures because in our retrospective review, patients with Bismuth type III and IV strictures who underwent bilateral stenting were short in numbers and had an incomplete follow-up with important data missing; therefore, they could not be included in the study. Furthermore we avoid attempting bilateral stenting in very advanced disease because in cases of complex hilar strictures bilateral stenting may not be sufficient for complete drainage. In these patients complete drainage is achieved by 3 or more metal stents and even successful bilateral stenting in advanced disease can leave some ducts filled with contrast undrained [33]. Injection of contrast into intrahepatic ducts that cannot be adequately drained should be avoided, as this practice is associated with worse outcomes [19]. A recent study by Costamagna group suggests that multiple SEMS can be placed in hilar malignant strictures with promising results and that SEMS malfunctions can be easily managed [34]. In our study using metal stents, bilateral placement was successful in 42/45 (94.3%) patients, and these results are comparable to those of other reports that found a TS rate between 80-100% [17,22,24-27]. The median stent patency time of our bilateral metal stenting was 29 weeks, which was similar to the 130 to 239 day range [17,22,24-27] reported by other authors, except for [Naitoh et al. 21], who reported a median stent patency of 488 days. We found a significantly lower occlusion rate with bilateral metal stenting (11.9% versus 31.4%) compared with unilateral SEMS placement. Our bilateral stent occlusion rate compares favorably to previously reported values of between 23 and 40% [22,24-27], although a recent study reported an occlusion rate of 6% [26].

We also found that bilateral PS stenting was significantly better than unilateral PS placement in terms of patency and occlusion rates. The differences in patency were small (median 17 weeks; mean 15 weeks for unilateral versus median 18 weeks; mean 18 weeks for bilateral) but statistically significant. In contrast to the present study, [De Palma et al. 20] did not report stent patency and occlusion rates. Furthermore, in the study of De Palma et al., the poor results of bilateral stenting from the ITT analysis with high rates of cholangitis and lower TS suggest that the opacification of both lobes was performed even in patients with advanced disease. As mentioned earlier in this paper we did not attempt to place 2 PS stents in patients with advanced disease (Bismuth III and IV strictures) where bilateral stenting may not be sufficient for complete drainage, leaving ducts undrained with associated cholangitis. Taken together, these findings suggest that bilateral stenting is better than unilateral stenting, and the best results are produced with bilateral metal placement especially in patients with Bismuth type II strictures.

We did not find significant differences in survival, although some of the patients with bilateral metal stenting had improved survival time especially those free from re-intervention and occlusion with cholangitis. Although cholangitis has a negative impact on survival time, survival depends mainly on the disease stage and we had more patients (49 vs. 72) with advanced disease (Group 3) in the SEMS group, and we can speculate that was the main reason why, in this study, SEMS placement did not translate into a survival benefit. A recent randomized trial comparing PS with SEMS has shown a survival benefit for patients treated with SEMS [13]. In our study patients treated with SEMS had a lower rate of re-interventions and complications and we can speculate that can be translated into a better quality of life. Furthermore, although a cost/benefit analysis was not done, the lower number of re-interventions, days at hospital and hospital re-admission found in patients submitted to SEMS placement suggests a clear benefit for SEMS and in future studies that type of analysis, along with quality of life improvement should be undertaken.

Several limitations of our study should be taken into account. This was a retrospective study from a single tertiary center. Bilateral stenting was only reported in patients with Bismuth type II hilar strictures. The study was performed over 15 years using different materials, guidewires, stents and techniques, and this variation could have affected the rates of TS and FS, especially considering the plastic stents used in the 1990s. We suggest that further studies with a prospective randomized controlled design are needed in which bilateral and multiple metal stenting (in complex hilar strictures and advanced disease) can be compared with unilateral placement of SEMS across all Bismuth classifications of hilar CCA in terms of TS, stent patency, need for re-intervention, complications and improvement of the quality of life. In these proposed studies, different techniques for bilateral metal stent placement should also be compared.

The strengths of our study are the large number of patients included, both overall and in the different Bismuth classifications, when comparing plastic with metal stents, as well as the examination of bilateral stenting using both plastic and metal stents in a large subset of patients, which allowed comparisons between the outcomes. To our knowledge, this is the largest series of endoscopic palliation of hilar CCA ever reported.

## Conclusions

In conclusion, our data suggest that endoscopic palliation of hilar CCA with SEMS should be considered the standard of care, as it offers higher technical and clinical success rates in an ITT analysis as well as lower complication rates, lower number of reinterventions and superior cumulative stent patency when compared with PS placement for all Bismuth classifications. The cumulative stent patency of bilateral SEMS or PS stenting was significantly higher than that of unilateral SEMS or PS stenting, with lower occlusion rates in Bismuth type II patients. In this group of patients, bilateral SEMS placement offered the best results in terms of cumulative stent patency and the need for repeat endoscopic biliary drainage resulting from stent occlusion.

## Competing interests

None relevant to this manuscript.

## Authors’ contributions

Conception and design of the study, performing endoscopies, collection of data, analysis and interpretation of data, critical revision of the manuscript and approval of the final draft submitted: ML; performing endoscopies, collection of data, analysis and interpretation of data, drafting the manuscript, and approval of the final draft submitted: JC.

## Pre-publication history

The pre-publication history for this paper can be accessed here:

http://www.biomedcentral.com/1471-230X/12/103/prepub
